# Detection of Volatile Organic Compounds as an emerging strategy for Parkinson’s disease diagnosis and monitoring

**DOI:** 10.1038/s41531-025-00993-2

**Published:** 2025-06-12

**Authors:** Ilaria Belluomo, Munir Tarazi, Nicholas P. Lao-Kaim, Yen F. Tai, Patrik Spanel, George B. Hanna

**Affiliations:** 1 Department of Surgery and Cancer, Imperial College London, Hammersmith Campus, Commonwealth Building, London, UK; 2 Department of Brain Sciences, Division of Neurology, Imperial College London, Hammersmith Campus, Commonwealth Building, London, UK; 3 Department of Neurosciences, Imperial College London, Charing Cross Hospital, Fulham Palace Road, London, UK; 4 J. Heyrovský Institute of Physical Chemistry of the Czech Academy of Sciences, Prague, Czech Republic

**Keywords:** Diagnostic markers, Prognostic markers, Biomarkers, Neuroscience, Diseases of the nervous system, Parkinson's disease

## Abstract

Growing evidence suggests that specific volatile organic compound (VOC) profiles may reflect key pathophysiological processes in Parkinson’s disease (PD), including alterations in the microbiome, metabolism, and oxidative stress. Identifying reliable VOC biomarkers could enable non-invasive tests for early diagnosis, disease monitoring, and therapy evaluation. This review examines VOC analysis in biological matrices such as breath, skin, and stool, outlining current research and future applications in PD. We evaluate analytical techniques based on sensitivity, specificity, and clinical applicability. Additionally, we classify VOCs identified in previous studies alongside their proposed biological origins. Special attention is given to short-chain fatty acids, produced by the gut microbiome, a novel target in PD research. Our findings highlight the need for larger cohort studies and standardized protocols to advance VOC-based diagnostics in PD. Understanding the interplay between VOCs and PD may facilitate biomarker discovery, enhancing non-invasive diagnostic strategies and personalized disease management.

## Introduction

Parkinson’s disease (PD) is a progressive neurodegenerative disease with a rapidly rising incidence and prevalence^1^. Currently, the diagnosis of PD is based on clinical symptoms, which typically manifest when the progressive accumulation of α-synuclein-rich Lewy body pathology and loss of neuromelanin-containing dopaminergic neurons in the *substantia nigra pars compacta* is already at a mid-to-late stage^2^. The development of early diagnostic strategies for PD is crucial, as it would allow for timely intervention, before irreversible neuron loss, ultimately enhancing the quality of life of people with Parkinson’s (PwP)^3^. [^123^I]FP-CIT SPECT, a routinely-used nuclear imaging marker of the dopamine transporter, can supplement the clinical evaluation with a significant impact upon treatment course^4^, yet is considered unreliable at distinguishing between different conditions with nigral degeneration^5^. Identification of novel biomarkers could lead to earlier and more accurate diagnoses, which may have positive consequences on patients’ quality of life, by permitting more timely access to both pharmacological and non-pharmacological management, as well as increasing the efficacy of novel neuroprotective or disease modifying interventions.

In the wide panorama of biomarker detection techniques based on metabolomics, analysis of volatile organic compounds (VOCs) is a novel, powerful tool. VOCs are a broad group of organic chemicals that have high vapor pressure and low water solubility which allows them to easily evaporate at room temperature^6^. Their volatility enables the development of non-invasive methods for their detection. VOCs are end-products or intermediates of cell and bacterial metabolic activity, and changes in cellular metabolism or microbiome composition can modify their production^7^. As such, altered VOC profiles can be an indicator of physiological changes or pathological states.

VOCs can be detected in various biological matrices, including exhaled breath, skin, stool, blood, and urine^8^. Recent technological advancements have enhanced the sensitivity and specificity of analytical methods, resulting in more reliable quantification and identification of these compounds, accelerating the creation of non-invasive diagnostic tests including breath tests^9^. Such VOC-based diagnostic approaches are particularly attractive for complex pathologies for which an earlier diagnosis and a start of therapeutic management significantly improves survival rates or patient quality of life. Since these tests are easy to perform and have a high patient acceptability^10^, in the future it will be possible to employ them for more accessible tests in primary care, completely revolutionising the medical diagnostics landscape.

However, further developments are required for the full adoption of VOC-based tests in the clinical practice. VOCs have high biological variability, being influenced by factors such as diet, medications, life-style and environmental exposure, making standardisation difficult^7^. The biological sample of choice requires careful collection protocols to minimise contaminations and ensure reproducibility. In addition, VOCs are present in very low concentrations in these biological samples, and the required highly sensitive and specific instrumentations may not always be practical for routine clinical use.

Despite the methodological challenges, clinical trials evaluating non-invasive tests based on VOC detection have yielded promising results in conditions such as cancer^11,12^ and respiratory disorders^13^. Nevertheless, their application within the field of neurology has been much less explored. Recent data, updated in May 2023^14^, showed that less than two percent of clinical trials using breath analysis were focused on neurological diseases. Despite this, interest in the involvement of VOCs in neurological diseases is slowly growing. Variations in the production of VOCs in breath, skin and stool, have already been documented in PwP compared to healthy controls^15^. Moreover, these changes have been linked to gut microbiome dysbiosis which is characteristic of PD, offering high mechanistic validity for their potential as early biomarkers^15^. In particular, stool content of short-chain fatty acids (SCFAs), a very important class of VOCs and end-product of microbial metabolism, is altered in PwP compared to controls^15^. SCFA depletion could be among the causes of the early non-specific gastrointestinal symptoms typical of PD, which can arise many years before the onset of motor symptoms^16^. Thus, an imbalance of VOCs could already be present in the prodromal phase of PD.

In this review, we aim to provide a comprehensive summary of all studies conducted to date in which VOCs have been measured in various biological samples in PD. We specifically focus on studies where the diagnostic role of VOCs as biomarkers has been explored. We examine the different analytical techniques employed for VOC analysis, including mass spectrometry and sensor-based approaches, and highlight the strengths and limitations of each method. Finally, we list and critically revise the proposed biological origin of each VOC, aiming to establish possible connections with pathways previously outlined in the literature.

## Results

### Analytical techniques for VOC detection in PD

Variation in VOC levels in PD have been explored across both pre-clinical and clinical studies with the aim of developing non-invasive tests for diagnosis and/or monitoring. The choice of the analytical technique for VOC measurement largely varies, depending on the type of sample to analyse and the application.

The most used analytical technique in these studies has been gas chromatography mass spectrometry (GC-MS), which currently represents the gold-standard for VOC detection. Gas chromatography is able to separate constituents of complex biological samples, such as breath, stool, or plasma, while the mass spectrometry component measures the mass-to-charge ratio of each compound and relative signal intensities. Given the physiological low concentration of volatiles, GC-MS is widely adopted in VOC analysis for its high sensitivity, allowing detection of trace amounts of substances. However, a number of limitations must also be taken into account for this technique. The limited mass range may restrict the detection VOCs with high molecular weight. Derivatisation is often required for the analysis of the less volatile or thermally labile compounds, adding complexity and potential variability to the workflow. Additionally, the analysis time can be lengthy, in some cases taking more than an hour per sample. Adsorption and desorption effects can influence results, such as a preferential desorption of certain analytes while others may be retained on sorbent materials, which can introduce biases in VOC profiles. High background noise from fibres or other sorptive materials can further complicate data interpretation.

Different sample introduction techniques, based on the nature of the sample to analyse, can be coupled with GC-MS. Among these, the most commonly used has been the thermal desorption (TD) unit, which analyses samples collected in TD tubes in an entirely automated fashion. TD tubes, stainless steel tubes containing a sorbent phase able to trap VOCs, are frequently used for both breath analysis and analysis of volatiles from other matrices. The sorbent phase can be made of different materials, depending on the chemical nature of the volatile compounds to analyse. They are robust, easy to transport and store, and they are therefore a precious tool in large-scale multi-centre clinical studies^17^. Breath can be collected onto tubes using different commercially available devices, or through bags^18^. Breath bags can be made of different inert materials, such as Nalophane or Tedlar, then transferred into TD tubes prior to GC-MS analysis. In the case of non-gaseous samples, such as stool, plasma, tissue, or gauze where skin sebum has been collected, headspace is generated by sample heating and then transferred into the tubes. A few studies have employed solid phase micro extraction (SPME) as a sample preparation technique, which uses a stationary phase coated fibre for the extraction of VOCs. The coated fibre adsorbs compounds of interest, and it can be directly introduced into GC-MS for analysis. Targeted methods can be designed for specific compounds of interest with GC-MS using acquisition parameters previously determined using chemical standards. Alternatively, untargeted approaches can be used when the identity of the compounds of interest is not known. In this case a tentative compound identification can be performed through online mass spectra library matching. However, this method can lack precision, and it is not always possible to confirm molecular structures with confidence. The identification of VOCs is often not univocal and challenging, leading in some cases to misidentifications^19^.

While GC-MS, despite the discussed limitations, remains the gold standard, different approaches are also emerging to enhance VOC detection and characterisation. Direct sampling techniques, such as proton transfer reaction mass spectrometry (PTR-MS), selected ion flow tube mass spectrometry (SIFT-MS) and secondary electrospray ionisation (SESI-MS) allow real-time analysis of VOCs, without requirements for sample pre-treatment, making them promising tools for dynamic metabolic studies^7^. Solvent extraction of fibres may enable VOC isolation for liquid chromatography-mass spectrometry (LC-MS) applications, broadening the range of detectable compounds beyond traditional GC-MS capabilities. These alternative methods may help address some of the challenges associated with standard VOC analysis techniques.

Breath represents one of the most readily accessible matrices for the measurement of volatile compounds, making it exceptionally well-suited for future large population screening. For this reason, diagnostic studies frequently focus on analysing breath VOCs, given its high potential for widespread screening. Nanotechnology-based sensors have been used in many studies with the aim of diagnosing PD from breath composition alterations. Sensors have numerous advantages, they can be miniaturised, having a high surface-to-volume ratio, therefore being highly sensitive to small changes in chemical compositions. In addition, they have a fast response and recovery time, thereby providing a quick measurement of dynamic matrices fingerprints. However, sensors can be limited by their specificity due to analyte cross-reactivity with other compounds present in breath or interference from environmental factors that may lead to false positive or inaccurate readings^7^. Sensors alone lack the capability of identifying or quantifying VOCs. They do not target specific biomarkers, but are designed to detect overall patterns in volatile compounds that distinguish between patients and controls. It provides a general indication of differences in the breath profile, allowing for classification of samples. GC-MS is therefore used as a complementary technique in most studies utilising this technology, helping to assess the chemical composition and identify the specific compounds responsible for the observed differentiation between the breath of PwP and controls identified by the sensors. In such cases, there is the possibility of GC-MS not confirming the same separation between disease group and controls^20^.

In a few studies, alternative VOC measurement techniques, such as nuclear magnetic resonance (NMR), have been used. NMR may have a lower sensitivity and selectivity than GC-MS, however, is highly reproducible and able to provide precious structural compound information. In addition, NMR can be used in studies in which less-volatile compounds need to be measured, such as amino acids^21^.

Although sensors are an attractive analytical approach for VOC detection, further technical advancements are needed to enhance their robustness and reliability. Unlike GC-MS, which provides precise identification and quantification of specific VOCs, sensors detect overall patterns of volatile compounds that differentiate between groups, such as PD and non-PD. This difference in approach makes GC-MS the current gold standard for biomarker discovery and validation, while sensors offer a promising but less specific alternative for classification. In addition, variability in sample collection protocols, data acquisition, and analytical workflows results in inconsistencies in reported VOC profiles. Establishing standardized guidelines for sample collection, instrument calibration, and data interpretation will be crucial for translating VOC research into clinical applications.

### Pre-clinical studies in PD animal models

Animal models of PD have been employed to investigate the utility of VOCs across many aspects of the disease, ranging from diagnostic approaches to therapeutic interventions. Only a few studies have used these models to explore VOCs as diagnostic biomarkers^22–24^. Within these studies, particular attention has been given to the role of VOCs as indicators of microbiome changes or key mediators of inflammation and oxidative stress, such as SCFAs and alkanes. SCFAs, primarily produced by the gut microbiota through the fermentation of dietary fibres, play an important role in maintaining gut health and modulating immune responses, while alkanes have been implicated in oxidative stress processes. During oxidative stress, the production of reactive oxygen species (ROS) leads to the oxidation of various compounds, which can result in the formation of alkanes. A more detailed discussion of VOCs, particularly those associated with the microbiome, is provided later in the manuscript.

### VOC as PD diagnostic biomarkers in animal models

All the pre-clinical studies focusing on the diagnostic potential of VOCs have utilised rats treated with the neurotoxic compound 6-hydroxydopamine (6-OHDA). 6-OHDA has been administered by unilateral stereotaxic injections into the lateral ventricle, resulting in selective lesioning to the dopaminergic nigrostriatal system, confirmed by HPLC analysis of striatal tissue^25^. In one of these studies, PD-like rat breath was compared with breath from a group of sham-operated rats. Breath was sampled through tracheal intubation, allowing the exhaled air to be transferred into a Tedlar breath sampling bag, and analysed with both a sensor array and GC-MS to identify compounds driving the study groups separation. Six VOCs were identified as being higher in the breath of 6-OHDA rats, five of which belonged to the chemical class of alkanes, with the addition of styrene. Alkane over-production was hypothesized to originate from oxidative stress-related damage and cell death^22^. Styrene is likely an exogenous compound, and the reason for its increased concentration in 6-OHDA rat breath is unknown. Blood and striatum VOCs were investigated in two further studies from the same group. Khatib et al. compared VOCs from 6-OHDA rats to both sham-operated controls as well as to those that had selective lesions of noradrenergic neurons created through injection of N-(2-chloroethyl)-N-ethyl-2-bromobenzylamine (DSP-4), with the aim of establishing whether degeneration of different neuronal populations produced specific patterns of VOC imbalance. SPME followed by GC-MS analysis revealed a number of compounds with different concentrations between the 6-OHDA and saline control groups in both biological matrices^23^. Interestingly, the seven VOCs significantly altered in the 6-OHDA rats were either elevated in blood or reduced in the striatum, and these changes appeared dissimilar to those observed for the DSP-4 group, in which less difference were found compared to controls. The compound 1-octen-3-ol, an alcohol terpene, was found to be altered in both blood and striatum, with blood levels positively correlating with brain dopamine. Different potential biological origins were hypothesised for this VOC, among which was oxidation of linoleic acid, and it is thought to have deleterious effects on dopamine homeostasis^23^. In a third study, rats treated with 6-OHDA (50% or 70% dopaminergic denervation) were compared to a transgenic rat model carrying the human SNCA A53T point mutation to mimic progressive α-synuclein pathology. Rats with serotoninergic denervation, derived by injection of 5,7-dihydroxytryptamine into the raphe nucleus, together with sham-operated rats, were also included as control groups^24^. The degree of dopaminergic neuronal loss, choice of PD induction and type of neuronal lesion were tested across three matrices (blood, striatal tissue and breath) to find specific VOCs with diagnostic potential for PD. Measurements of breath VOCs obtained using sensors were analysed with multivariate statistics and showed good accuracy in differentiating between groups. Four VOCs were found to be significantly higher in the blood of the transgenic rats compared to the controls, but two of them were not identified due to low concentrations, while in striatal tissue one compound was elevated and three were completely absent^24^. The biological origin of most of the identified compounds is similarly theorised to be linked to oxidative stress. Unfortunately, GC-MS results for breath were not published due to a technical failure. The reliability and repeatability for the sensor arrays used in these few pre-clinical studies, despite obtaining a good separation among study groups, were not confirmed nor translated in further studies. All pre-clinical studies in which VOCs have been investigated as diagnostic biomarkers are summarised in supplementary data S1.

### VOC as PD monitoring biomarkers in animal models

Several other studies have utilised the ability to measure VOC production as a tool to investigate and monitor PD-related changes in the microbiome. Of particular importance is the monitoring of SCFAs following therapeutic interventions in studies whereby new compounds intended to ameliorate dysbiosis were tested. In contrast to the previously discussed diagnostic studies, mice were mostly used instead of rats, and 1-methyl-4-phenyl-1, 2, 3, 6-tetrahydropyridine (MPTP), a precursor of the MPP+ neurotoxin able to damage and permanently destroy dopaminergic neurons^25^, was more often used to induce PD-like disease. Two studies evaluated the effect of faecal microbiota transplantation (FMT) in mice on dysbiosis and PD symptoms. FMT is potentially able to restore a healthy balance in the gut microbiome, and for this reason could represent a promising novel therapeutic intervention for PD^26^. In both studies, FMT improved motor dysfunction and provided some restoration of dopaminergic neuron numbers. Measurement of stool SCFA content - acetic acid, propanoic acid, butyric acid and valeric acid – by GC-MS, revealed higher levels in MPTP mice as compared to controls at baseline, while after treatment SCFA levels decreased and became comparable to the control group^27,28^. In one of these studies, bacteria characterisation was also performed, and the dysbiosis present in the MPTP mice was alleviated for a number of taxa by the FMT intervention, such as *Firmicutes, Proteobacteria* and *Clostridiales*^27^. Molecular mechanisms were also investigated in both studies. In the first study, TNF-α, TLR4, NF-kB and TBK1 expression levels in both mouse colon and striatum that were heightened as a result of MPTP lesioning showed some alleviation following FMT treatment, suggesting that the reversal of gut dysbiosis may reduce both gut inflammation and neuroinflammation through suppression of the TLR4/TNF-α signalling pathway^27^. Similarly, in the second study, FMT was found to also inhibit the expressions of p-PI3K, p-AKT, TLR4, NF-kB and TNF-α that constitute a signalling cascade associated with microgliosis, thus providing evidence of a potential link with SCFAs, dysbiosis and the pathogenesis of PD^28^.

A number of other studies have investigated the effects of changes in dietary regimen or supplementation on microbiome composition. Zhou et al. found that MPTP-mice who had been subjected to a fasting-mimicking diet exhibited most SCFA levels that were more similar to the control group than the untreated MPTP group. Moreover, the intervention produced decreases in neuroinflammatory mediators, such as TNF-α and IL-1β, and an increase of brain-derived neurotrophic factor (BDNF), that promotes survival of dopaminergic neurons^29^. Interestingly, in contrast with the previous two studies in which the levels of all SCFAs were increased in the stool of the PD-like group compared to control group, in this study propanoic acid was lower in the PD-like group, while acetic acid was equivalent in the two study groups with no intervention^29^. Concentrations of total faecal SCFAs and butyric acid were also significantly lower in the PD-like group compared to controls in a study investigating prevention of dopaminergic neuron loss through administration of a seaweed polysaccharide, the polymannuronic acid (PM). The administration of this compound caused the increase of total SCFAs, acetic acid, propionic acid, and butyric acid, together with improving motor function in MPTP-mice^30^. Similarly in another study chicoric acid was also proven to have a neuroprotective power preserving dopaminergic neuronal death and motor dysfunctions, restoring eubiosis and SCFA levels^31^. In the MPTP group, levels of all SCFAs were significantly higher compared to controls and decreasing in the MPTP-group treated with two different doses of chicoric acid (30 or 60 mg/kg). The pre-treatment with vancomycin also showed protective effect on MPTP mice in a similar study^32^. Microbiome composition and SCFA levels were investigated in the presence or absence of the pre-treatment. High levels of butyric, valeric, and isovaleric acids were found in MPTP untreated mice compared to controls. The levels of these SCFAs decreased thanks to the treatment, showing comparable levels to the control group. The treatment did not change levels of propionic, acetic and isobutyric acids^32^.

Restored production of propionate through microbiome modulation was hypothesised to be the underlying mechanism of action for the PD-protective effect exerted by osteocalcin, a protein secreted by osteoblasts^33^. This result was further confirmed by finding a direct protective effect after propionate oral administration, finally confirming the modulating role of this acid in the osteocalcin mechanism of action. Interestingly, propionate was found to be lower in the faecal samples of 6-OHDA mice, but not in their serum. Butyrate and acetate were not altered in the 6-OHDA mice compared to controls^33^. The only study using a different animal model was the one by Yan et al., recreating early PD symptoms in a cohort of monkeys through A53T transgenic modifications^34^. In this study microbiome population differences between the A53T group and controls were investigated, as well as metabolite production, to find alterations already present in the early phases of the disease. In contrast to previous work, no significant alterations of SCFAs were found in the A53T cohort^34^. All the pre-clinical studies discussed above in which SCFAs have been investigated as PD-related biomarkers for disease monitoring are summarised in supplementary data S2.

### Clinical studies: VOCs as PD diagnostic biomarkers

**Fig. 1:**
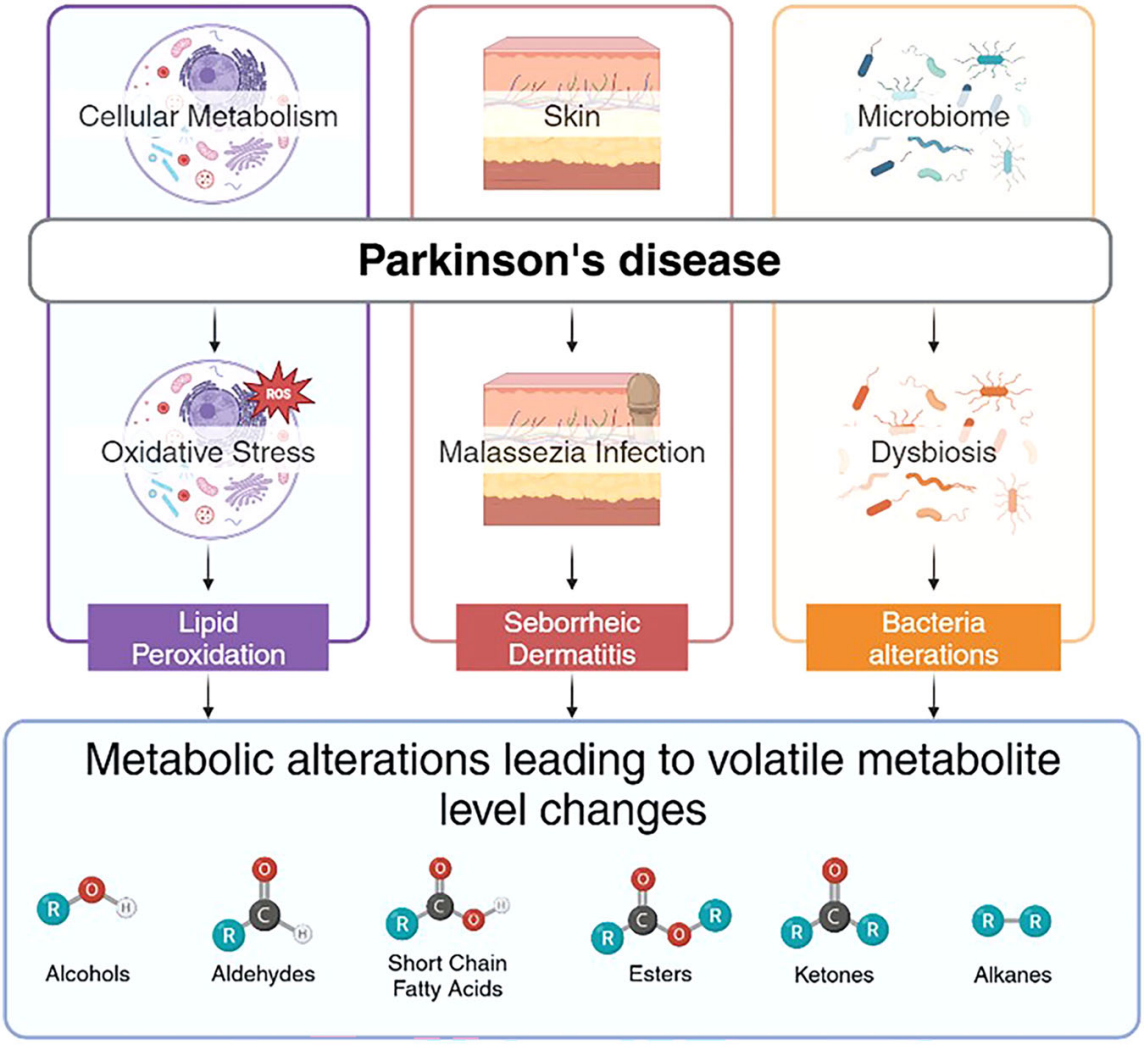
Schematic representation of the clinical studies. Different biological origin proposed in literature studies for volatile organic compounds (VOCs) related to pathological changes caused by Parkinson’s disease. Breath VOCs potentially originate from changes of cellular biochemical processes linked to oxidative stress. PD patients present in some cases skin infections, likely related to *Malassezia* yeast, that may be able to change the local VOC production. Gut dysbiosis is also a potential cause of VOC changes, since different bacteria produce different VOCs. In the lower section of the figure, the most common identified volatile metabolite chemical classes originating from these processes. Figure created in https://BioRender.com.

The development of a non-invasive test is very attractive for population screening and early diagnosis of PD. VOCs contained in exhaled breath and emitted by skin have been investigated in some recent clinical studies to find a specific diagnostic signature towards this aim. VOCs originating from the gut microbiome have been explored in humans, as well as in animals as described in the previous paragraph, as a product of the dysbiosis related to PD. A schematic representation of the clinical studies and their rationale is shown in Fig. 1.

#### Breath

As mentioned above, breath VOCs represent an attractive opportunity for the development of non-invasive tests for early PD diagnosis, which would be acceptable by patients and feasible on a large-scale. In some studies, PD has been considered for the development of a non-invasive diagnostic test alongside Alzheimer’s disease (AD)^35–37^, pathologies that have certain similarities in terms of symptoms, particularly in their early stages, and a comparable age of onset^38^. For both neurodegenerative disorders, the antemortem diagnosis relies on clinical criteria only, and there are still no reliable biomarkers indicating their onset and development. Given their gradual pathological progression, patients might show sub-clinical biochemical changes many years before clinical symptoms appear. It is on this premise that the development of breath diagnostic tests based on VOC detection is founded. In two studies, compounds associated with PD or AD that differentiated them from healthy controls were identified using GC-MS, after group separation had previously been obtained with sensor-based analysis^35,36^. In both diseases, some compounds including aromatic hydrocarbons and alkanes had a higher breath concentration as compared to healthy controls, with the authors proposing oxidative stress as a possible origin^35^. Both studies also identified siloxane compounds, specifically decamethyl-cyclopentasiloxane^35^, cyclopentasiloxane and cyclotrisiloxane^36^, as PD-specific biomarkers. However, a cautious approach must be adopted as to the speculation of a biological role for these compounds, since they could be contaminants originating from the GC-MS analytical columns^39^. With a different approach involving the use of ion mobility mass spectrometry (IMS) for compound identification, Bach et al. demonstrated that the alcohol 1-butanol was able to discriminate PD from both AD and HC in the first classification of a decision tree data mining model, with 100% accuracy^37^.

Similarly to AD studies, breath content was measured with an array of nanomaterials to distinguish idiopathic PD from other parkinsonian syndromes^40^, based on results from a previous pre-clinical study^22^. Currently, differential diagnosis between PD and other parkinsonian disorders can be challenging, involving a comprehensive evaluation of clinical symptoms, medical history, response to medications and additional diagnostic tests, such as neuroimaging^41^. Four VOCs were identified through GC-MS complementary analysis that were able to discriminate idiopathic PD from a non-specific Parkinsonism group. The authors hypothesized that these compound alterations could be due to several biological processes, among which oxidative stress, cytochrome P450 effect, hepatic enzymes and carbohydrate/lipid metabolism have been suggested^40^. Following this, the same group conducted a large multi-centre study in which a single nanoarray sensor was constructed and tested for its ability to detect and classify between 17 different disease states categorised as either cancerous, inflammatory or neurological, which included both PD and atypical Parkinsonism, as well as multiple sclerosis^42^. The study involved the analysis of 2808 breath samples collected across 9 clinical centres worldwide, with GC-MS analysis also conducted to support the results of the nanoarray and to identify discriminating compounds. A heat map was reported with the levels of thirteen VOCs associated in different measures with the 17 pathologies examined. For PD, 3-methylhexane and nonanal showed a lower concentration, while, in agreement with a previously discussed paper from the same research group, styrene was elevated^42^. More recently, Stott et al. demonstrated that as well as discriminating patients from controls, nanoarray sensors were also able to separate patients with a disease duration above or below 6 years with 81.5% accuracy. Further complementary analysis using GC-MS identified candidate VOCs including propanal, acetone, pentane and isoprene that were elevated in the more as compared to less advanced patients (Hoehn & Yahr > 3 or Addenbrooke’s cognitive exam < 90). Pentane in particular, which survived significance correction in both analyses, was hypothesised by the authors as a possible marker of oxidative stress secondary to alpha-synuclein pathology^43^. Notably, in line with the results of a similar prior assessment concerning levodopa and MAO-B inhibitors^40^, the authors were also able to show that there was no significant confounding influence of levodopa treatment on the sensor-based classification, an important result given the likelihood for medications to affect metabolic processes and thus alter the production of endogenous volatiles. Building on this, Finberg et al. limited recruitment to only early-stage *de-novo* patients who had not yet received any drug-related or non-pharmacological treatment, still finding good classification sensitivity, specificity and accuracy through use of a sensor array (79%, 84%, and 81% respectively)^20^, helping to support the use of the volatilome as an early diagnostic biomarker. However, despite the results obtained with the sensor technology, GC-MS analysis was not able to detect a significant difference between groups. Though sub-threshold, 11 selected VOCs appeared to be elevated in PD patients as compared to controls, among which benzaldehyde exhibited the most prominent difference between the two groups (*p* = 0.08, Wilcoxon nonparametric test). According to the authors, the relative differences among these 11 compounds, despite not being individually significant, collectively create the altered VOC spectrum detected by sensors. All the VOCs increased in the breath of PwP compared to controls, with a possible endogenous metabolic origin are listed in Table 1.

**Table 1:** Volatile biomarkers increased in breath

Ref.	VOC	Chemical class
Tisch et al.^35^	2,3,6,7-tetramethyl-octane	Alkanes
	butylated hydroxytoluene	Phenolic compounds
	5-ethyl-2-methyl-octane	Alkanes
Nakhleh et al.^40^	3methylhexane	Alkanes
	2-pentanone	Ketones
	benzaldehyde	Aromatic hydrocarbon
	acetophenone	Aromatic ketone
Finberg et al.^20^	2,3-dimethylpentane	Alkane
	Acetophenone	Aromatic ketone
	Benzaldehyde	Aromatic hydrocarbon
	beta-Myrcene	Terpene
	Undecane	Alkane
	Nonanal	Aldehyde
Stott et al.^43^	Pentane	Alkane
	Propanal	Aldehyde
	Acetone	Ketone

Volatile compounds increased in the breath of people with Parkinson compared to healthy controls with a possible endogenous metabolic origin.

#### Skin

VOCs emitted from skin are derived from sweat or sebum, produced by sebaceous glands, or generated by bacteria present on the skin surface^44^. The continuous exposure to the external environment makes the identification of specific skin endogenous VOCs and their biological interpretation very challenging. However, skin has always been considered an interesting matrix to explore for volatile biomarker research, since it is easily accessible and offers the future possibility of developing wearable devices. Increased sebum production, known as *seborrhoea*, is a well-documented non-motor symptom of PD, and is thought to result from autonomic dysfunction affecting sebaceous gland activity. This alteration can contribute to skin conditions such as seborrheic dermatitis, which is more prevalent in PD patients. The changes in sebum composition and production may also influence the VOC profile of the skin, making it a relevant factor in PD biomarker research. The research about skin volatile compounds related to PD had a breakthrough from the unique ability of Joy Milne to detect the odour of the disease. Her husband was diagnosed with PD, and some time before the confirmed diagnosis she noticed his scent had changed. Her ability was tested in a pilot study in which she blind smell-tested t-shirts from PD patients and controls. She misclassified just 1 t-shirt from the control group, only to then find out that this participant received a PD diagnosis soon after^45^. The story of Joy Milne demonstrated the existence of an odour cue from the skin of PwP. She collaborated with a VOC research group as a “Super Smeller”, trying to identify the precise chemical volatile signature that gives the characteristic smell that she is able to detect. In a first study, sebum was collected from 64 participants in total, consisting of medicated PwP, drug-naïve *de-novo* PwP, and healthy controls, and divided into a discovery and validation cohort. VOCs were collected by swabbing the upper back using medical gauze and were then measured using GC-MS^46^. A PLS-DA model identified 17 metabolites discriminating PD in the discovery cohort, 9 of which were confirmed in the validation cohort. Further univariate analysis together with fold change calculations showed that 4 of the 9 identified VOCs had a similar alteration between the two cohorts. This study also emphasised the importance of including a *drug-naïve* group in the study design. Specifically, 3,4-dihydroxy mandelic acid was identified as altered in the PD group. This compound is a metabolite of L-dopa, a dopamine precursor commonly and effectively administered as a dopamine replacement therapy for PwP. However, it was also observed in both the *drug-naïve* and control groups, indicating its potential endogenous origin^46^. A series of odour tests were also performed by the Super Smeller, smelling chemical compounds isolated through GC-MS analysis using an odour port. She identified the region of the chromatogram in which she recognised the characteristic PD-related smell; this region contained 3 of the compounds of interest previously identified: hippuric acid, eicosane and octadecanal. A targeted study to validate these findings in a new PD cohort was carried out a few years later by the same group, together with a further untargeted screening of skin VOCs^47^. Similar to the first study, the participant population was composed of a *drug-naïve* PwP group, together with treated PwP, and controls, to exclude any effect of medications on biomarker identification. A good separation was obtained between all PwP and controls (correct classification rate, CCR: 88.4%), while the measured VOC profile did not reliably discriminate *drug-naïve* and medicated PD (CCR: 66.7%), providing some evidence that medication exerts minimal influence. Eight compounds, all decreased in the PD group, were identified by the discriminating model. However, compounds annotation could not be performed due to the poor performance of the database matching to available spectra libraries. The annotation issues made the validation of the VOCs identified in the previous study not possible^47^. Sebum composition was also investigated in another study, in which GC was combined with a surface acoustic wave sensor, in combination with a machine learning developed algorithm. Three skin biomarkers were found to give a good separation between PD and controls: octanal, probably originating from oxidative stress, hexyl acetate, and perillic aldehyde, likely related to seborrheic dermatitis, a skin disease affecting PwP^48^. All the clinical studies examining both breath and skin, with the listed compounds and proposed biological origin are listed in supplementary data S3.

### VOCs as products of microbiome

The role of the gut microbiome in VOC production in physiological and pathological conditions is well documented. A growing body of evidence suggests that many VOCs, especially SCFA, are produced by the gut microbiome, which is known to be significantly altered in PD, making it a crucial factor to explore when examining the role of VOCs in this pathology.

The literature search identified five papers that reported on bacterial changes in the stool of PD and their association with VOC production^15,49–52^. Heterogeneity was identified in the microbiome assessment methods employed among the studies with one study using qPCR to quantify genes targeting specific bacteria^15^. Three studies used amplicon targeted sequencing, namely the V3-V4 variable region of the 16S rRNA gene^49–51^ and one study employed shotgun metagenomic sequencing^52^.

Four out of the five studies measured α diversity, the variety of microbial species within a single sample, and β diversity, the microbial composition between different samples. α diversity measures the richness (number of species) and evenness (distribution of species) within a single sample or community. This allows assessment of the local diversity of microbial species within the sample. Three studies reported both an increase in α diversity and a shift in β diversity in PD as compared to controls^50–52^, while one reported no significant change for either metric^49^. Furthermore, Aho et al. demonstrated that α and β diversity were associated with severity of PD symptoms as measured by the Hoehn & Yahr and UPDRS scales, as well as motor subscores rating the degree of tremor and akinesia/rigidity^51^. In relation to difference in bacteria between PD and controls, there was no consensus in the published literature.

Aho et al demonstrated an enrichment of *Bifidobacterium* in PD compared to an enrichment of *Bacteroides* in healthy controls, a known SCFA producing bacteria. This could explain the positive association between SCFAs in controls, and a reduction in PD patients. *Bifidobacterium*, normally considered to be butyrogenic due to cross-feeding interactions with butyrate-producing bacteria, showed a negative association with butyric acid in PD, which could indicate a deleterious change in its homeostatic functions and/or strain profile. This is further supported by a positive correlation with the inflammatory marker, neutrophil gelatinase-associated lipocalin, that is not present in the control group. Other SCFA-producing taxa such as *Phascolarctobacterium* similarly evince disparate associations commensurate with a change in metabolic function^51^. Later, Chen et al. demonstrated that faecal acetic, propionic and butyric acids all positively correlated with the abundance of *Bacteroides sp AM16-15* and *Bacteroides sp AM25-34* exclusively in the control group. Conversely, only in PwP were decreased faecal and increased plasma SCFA levels, especially that of propionic acid, correlated with abundance of proinflammatory microbes such as *Clostridiales bacterium NK3B98* and *Ruminococcus sp AM07-15*^52^.

Using analytical approaches such as phylogenetic placement or shotgun metagenomics to infer metabolic structure from microbial communities has highlighted a number of functional pathways predicted to have been altered in the Parkinsonian condition. Using *16S rRNA* gene sequencing data, Tan et al. predicted downregulation in pathways of choline biosynthesis and bile acid degradation, alongside upregulated glucosylglycerate biosynthesis and salicylate degradation, the latter of which interestingly possesses neuroprotective properties^49^.

This cohort was also observed by ^1^H-NMR to have reduced faecal butyrate, though direct associations between VOCs and pathways were untested. Furthermore, others have identified microbiota-related pathways including those related to peptidoglycan maturation, guanosine nucleotides biosynthesis, L-aspartate and L-asparagine biosynthesis and glycolysis IV that correlate positively with SCFAs in PD, especially propionic acid^52^. However, it is unclear whether these correlations are unique to the Parkinson’s state, or whether they also constitute a change in the normal metabolic functions of gut bacteria.

Ren et al. compared microbial gene function and cognitive impairment in PD^50^. PD with mild cognitive impairment compared to healthy controls were found to have higher microbial gene functions related to membrane transport including transporters, ATP-binding cassette (ABC) transporters, transcription factors, and benzoate degeneration in the level 3 KEGG pathway. When comparing PD with no cognitive impairment, PD had higher microbial gene functions related to glycerophospholipid metabolism, base excision repair, and signal transduction mechanism in level 3 KEGG pathway.

Plasma propionic acid levels were associated with PD occurrence and with motor symptom severity. *Ruminococcus sp AM28-29LB* consistently correlated with reduced faecal levels of all SCFAs and increased plasma butyric acid which was associated with cognitive decline in PD^52^. Taking all the above findings together, these indicate that alterations in microbial species abundance with alterations in related metabolic pathways area associated with plasma and faecal levels of different SCFAs, which can correlate with either cognitive or motor symptom severity in PD.

## Discussion

In this article, we reviewed the existing literature to provide an overview of the current evidence on VOC research in relation to PD. The reviewed studies highlight a growing interest surrounding the role of volatiles in PD for disease diagnosis and monitoring of both its progression and response to novel therapeutic interventions.

The detection of misfolding α-synuclein in the cerebrospinal fluid (CSF) of PwP using seed amplification assay has recently been shown to be one of the earliest biological signatures of PD^53^. However, the assay is binary, rather than quantitative, at present, and therefore cannot be used as a marker of disease progression. Additionally, obtaining CSF through lumbar punctures is relatively invasive, and may not be readily accepted by the wider PD community outside of enthusiastic research participants. Developing a non-invasive and quantitative method of detecting VOCs could potentially lead to a marker of disease progression in PD, which is currently lacking and would be hugely useful in disease modification studies. According to the ‘body-first’ hypothesis of α-synuclein origin and propagation, the pathology of PD in some patients starts in the peripheral nervous system, most likely the enteric nervous system^54^. The gut dysbiosis which can lead to changes in VOCs of PwP may be detectable even before the presence of misfolding α-synuclein in the CSF. Such early detection may be important if we have an effective disease modifying intervention available in the near future.

In clinical studies, breath and skin are the most attractive matrices for the development of early non-invasive diagnostic tests. Different analytical methods have been employed to measure VOC levels and identify specific profiles related to PD. Sensor arrays offer a convenient and rapid approach to detecting pathological VOC signatures associated with PD in breath. GC-MS has been commonly used to identify compounds responsible for the group separation obtained by sensors. However, it is challenging to directly correlate the results of these two techniques due to differences in sample collection, handling, and analysis, which can introduce numerous biases. While GC-MS is a widely used technique for VOC analysis, its ability to detect non-derivatized compounds is inherently limited. Many polar or high-molecular-weight metabolites have low volatility and poor ionization efficiency, making their direct detection challenging. Functional groups such as hydroxyl, carboxyl, and amine groups often require derivatization to enhance volatility and improve chromatographic separation. As a result, certain biologically relevant metabolites may be underrepresented in GC-MS datasets, necessitating complementary analytical approaches for a more comprehensive characterization of metabolic pathways.

Deep profiling studies using high-resolution GC-MS are needed to have a certain degree of confidence in the identity of compounds. Once an agreement about the identity is reached, more convenient targeted methods can be developed to achieve large population screening. Sensors are expected to play a key role in the future of non-invasive testing; however, further technical advancements are necessary to ensure their analytical performance.

Another significant limitation of the clinical studies reported to-date is the low number of samples/subjects, and thus the inadequate accounting of heterogeneity within the patient population. Relatedly, control groups were not always appropriately matched to PD groups. Breath content can be influenced by many factors, such as diet or environment, and these have not always been taken into account, and the way to normalise these factors were not always explained. Large-scale, multi-centre studies with appropriately control selection are required to establish the true potential of volatile compounds in early PD diagnosis.

Many of the clinical and pre-clinical studies with a focus on diagnostic identified alterations in compounds likely associated with oxidative stress. However, there is no consensus on the specific identities of these compounds, and the results of these studies have not been further investigated to validate the findings. None of the identified volatile biomarkers have been tested for sensitivity or specificity in diagnosing PD in a large cohort, nor to establish a direct biological link in the PD pathogenesis or progression. Oxidative stress is related to PD and likely plays an important part in the production of potential biomarkers which could be used in early diagnostic models. However, the compounds found need to be validated to properly establish a direct biochemical link between compound alteration and pathology.

**Fig. 2:**
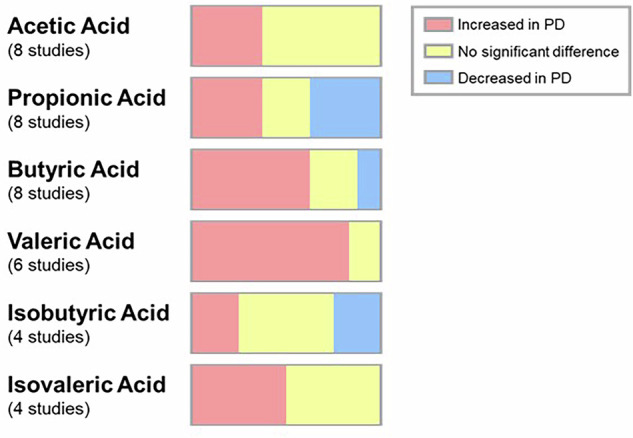
SCFA level alterations in Parkinson’s animal model stool samples. Alterations of SCFAs have been observed in PD mouse models compared to controls, as measured across various studies. Significant differences in SCFA concentrations have been demonstrated, providing insights into the potential impact of gut microbiome changes on PD pathology. However, there is no consensus about the fluctuation of their levels, as some acids have been found increased in some studies, decreased in others, or not changed.

SCFAs are of particular interest, given their role as potential mediators of PD-related neuroinflammation, described in many pre-clinical studies. Yet the lack of consensus on fluctuations in SCFA concentrations in the stool of PD animal models stands as a significant limitation within the literature thus far (Fig. 2). Reported results have been variable, despite the use of similar animal models and the same PD induction strategies, with MPTP being the most common in these studies. SCFAs are products of the microbiome and, as such, their altered levels come from an alteration of the microbial populations in the gut. The inconsistency in their levels between PD and controls is perhaps unsurprising then, given the similar lack of agreement regarding altered PD-specific microbiome populations^55^. One of the main reasons for this is that most microbiome sequencing undertaken uses amplicon sequencing which allows bacterial identification down to the genus level with only predicted functional pathways able to be identified. The gut microbiome is a dynamic environment which contains considerable redundancy, meaning different bacteria perform similar functions and fill the same ecological niche resulting in the same overall effect on cellular functions. Novel studies should clarify the alteration of bacteria related to PD, in terms of bacterial function and potential biomarker production. This can only be achieved by utilising deep metagenomic sequencing to identify bacterial strains and functions, along with culture-based mechanistic studies to examine the specific metabolic pathways involved in PD. The impact of bacterial alterations has been highlighted by the rise of probiotics as a potential adjuvant therapeutic intervention for PD that modifies the production of SCFAs through microbiome modulation^56^. As dysbiosis may begin many years before the emergence of motor symptoms^55^, it must remain an important avenue of investigation in pursuit of biomarkers that enable earlier detection and treatment for people with PD.

In conclusion, quantitative analysis of VOCs in several biological matrices, such as breath, skin, or stool, holds promise for developing non-invasive tests for PD. It is likely that the complexity of volatile biomarker alterations in PD originates from a combination of factors, rather than being a simple dichotomy between PD and healthy individuals, which may explain the current disagreement in findings. A deeper understanding of the interplay between these factors could lead to more accurate identification of volatile biomarkers and better diagnostic and monitoring tools for PD in the future. Methodological considerations are critical in volatile biomarker discovery and quantification. Large-scale clinical studies with independent validation trials are essential to gain the confidence required to introduce these tests in clinical practice.

## Methods

### Literature search and study selection

This systematic review was conducted according to the Preferred Reporting Items for Systematic Reviews and Meta-Analyses (PRISMA) statement. The systematic review protocol was registered on PROSPERO (CRD42024549014). Two authors (IB, NPLK) independently searched the following electronic databases: MEDLINE/PubMed (1966 to April 2020), EMBASE (1980 to April 2020), and the Cochrane Central Register of Controlled Trials (CENTRAL) from The Cochrane Library (2020, Issue 4) on the 23 January 2024. The search strategy for this review was constructed for each database by using a combination of medical subject headings (MeSH) and free-text terms, as shown in supplementary data S4.

Reference lists of selected articles were also examined to identify relevant studies that were not identified in the database searches. In addition, a search of the World Health Organization International Clinical Trials Registry, ClinicalTrials.gov, ISRCTN Register and PROSPERO databases was conducted to identify any ongoing or unpublished studies, but none were found.

#### Study selection

The inclusion criteria included studies describing VOCs and microbiome associations in PwP, randomised controlled trials (RCTs) and comparative observational studies in humans and animal models. Exclusion criteria included non-English studies, studies that did not include volatile organic compound profiling, conference abstracts, editorials, expert opinions, case reports, and non-comparative studies.

Two authors (IB, NPLK) independently reviewed all studies identified by the search strategy. After removing duplicates, the titles and abstracts of the studies were screened for inclusion using Rayyan software^57^. Where there was uncertainty from the study abstract, the full paper was assessed for relevance. Conflicts were resolved through discussion and involvement of a third author (MT) where necessary.

#### Data extraction and outcome measures

Two authors (IB, NPLK) independently extracted data from the included studies using an electronic data extraction spreadsheet. Disagreements were resolved through discussion and where consensus could not be reached, a third independent author (MT) was consulted. The primary outcome was the identification of reported associations of VOCs in PD. The secondary outcome was the identification of changes in the microbiome and their role in VOC production.

#### Study selection and characteristics

**Fig. 3:**
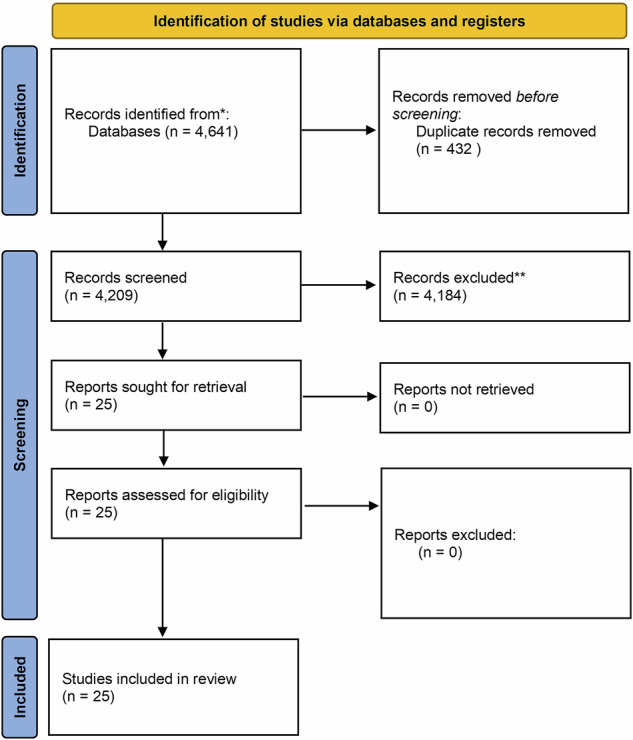
Prisma flow diagram for study inclusion. The figure presents the PRISMA flow diagram illustrating the process of study selection for the systematic review. The diagram outlines the number of literature records identified, screened, assessed for eligibility and finally included in the review, along with the reason for exclusion at each stage.

The literature search identified 4641 studies. After removing duplicates, 4209 titles and abstracts were assessed for eligibility. Following this screening, 4184 studies were excluded having not met the criteria for inclusion. The full articles of the remaining 25 studies were assessed with all being found to meet the inclusion criteria. A PRISMA flowchart^58^ summarising the search strategy is demonstrated in Fig. 3, while PRISMA checklist is in supplementary data S5. All included studies were observational studies, 11 were animal studies. All 25 papers reported on VOC profiles in PD and five papers reported on the changes in the gut microbiome linked to VOC production.

All studies were published between the years 2011 and 2022. 10 studies were undertaken in China, 8 in Israel, 2 in United Kingdom, the remainder were undertaken in Finland, USA, Canada, Germany and Korea. Across all included human studies, a total of 784 patients with PD were recruited.

## Supplementary information

Supplementary Information

Supplementary Data

## Data Availability

Data sharing is not applicable to this article as no datasets were generated or analyzed during the current study.
