# Supplementary material for: Detection of Volatile Organic Compounds as an emerging strategy for Parkinson’s disease diagnosis and monitoring
Source: NPJ Parkinsons Dis. 2025 Jun 12;11:161. doi: 10.1038/s41531-025-00993-2 (PMC12162845; doi:10.1038/s41531-025-00993-2)
Supplement: Supplementary file 1 — Supplementary Information [file 41531_2025_993_MOESM1_ESM.pdf]

Supporting Information

Detection of Volatile Organic Compounds: an emerging strategy for the diagnosis and monitoring of Parkinson's disease

Ilaria Belluomo[1*], Munir Tarazi[1], Nicholas P Lao-Kaim[2], Yen F Tai[3], Patrik Spanel[1,4], George B Hanna[1]

[1]Department of Surgery and Cancer, Imperial College London, Hammersmith Campus, Commonwealth Building, W12 0NN, London, United Kingdom.

[2]Department of Brain Sciences, Division of Neurology, Imperial College London, Hammersmith Campus, Commonwealth Building, W12 0NN, London, United Kingdom.

[3]Department of Neurosciences, Imperial College London, Charing Cross Hospital, Fulham Palace Road, London W6 8RF

[4]J. Heyrovský Institute of Physical Chemistry of the Czech Academy of Sciences, 18223 Prague, Czech Republic.

Supplementary Data 1. Pre-clinical diagnostic studies.

Summary of all the pre-clinical studies in which volatile compounds have been examined as diagnostic biomarkers.

Supplementary Data 2. Pre-clinical disease-monitoring studies.

Summary of all the pre-clinical studies in which short chain fatty acids have been measured to monitor Parkinson's progression after therapeutic interventions.

Supplementary Data 3. Clinical studies.

Summary of all the clinical studies in which volatile compounds have been examined as diagnostic markers.

Supplementary Data 4. Search Strategy.

Total results were 2562 - after duplicates: 2130. The search was performed on 23/01/2024.

Supplementary Data 5. PRISMA Checklist.

PRISMA 2020 checklist completed for the reporting of this review, indicating where each item is addressed in the manuscript.

Supplementary Data 5

PRISMA 2020 Main Checklist

**TITLE**			
Title	1	Identify the report as a systematic review.	Title, Page 1
**ABSTRACT**			
Abstract	2	See the PRISMA 2020 for Abstracts checklist	
**INTRODUCTION**			
Rationale	3	Describe the rationale for the review in the context of existing knowledge.	Introduction, Page 1-4
Objectives	4	Provide an explicit statement of the objective(s) or question(s) the review addresses.	Introduction, Page 4
**METHODS**			
Eligibility criteria	5	Specify the inclusion and exclusion criteria for the review and how studies were grouped for the syntheses.	Methods, Study selection, Page 19
Information sources	6	Specify all databases, registers, websites, organisations, reference lists and other sources searched or consulted to identify studies. Specify the date when each source was last searched or consulted.	Methods, Literature Search and Study Selection, Page 19
Search strategy	7	Present the full search strategies for all databases, registers and websites, including any filters and limits used.	Methods, Literature Search and Study Selection, Page 19
Selection process	8	Specify the methods used to decide whether a study met the inclusion criteria of the review, including how many reviewers screened each record and each report retrieved, whether they worked independently, and if applicable, details of automation tools used in the process.	Methods, Study selection, Page 19

(continued)

Data collection process	9	Specify the methods used to collect data from reports, including how many reviewers collected data from each report, whether they worked independently, any processes for obtaining or confirming data from study investigators, and if applicable, details of automation tools used in the process.	Methods, Data extraction and outcome measures, Page 19
Data items	10a	List and define all outcomes for which data were sought. Specify whether all results that were compatible with each outcome domain in each study were sought (e.g. for all measures, time points, analyses), and if not, the methods used to decide which results to collect.	Methods, Data extraction and outcome measures, Page 19
	10b	List and define all other variables for which data were sought (e.g. participant and intervention characteristics, funding sources). Describe any assumptions made about any missing or unclear information.	Methods, Data extraction and outcome measures, Page 19
Study risk of bias assessment	11	Specify the methods used to assess risk of bias in the included studies, including details of the tool(s) used, how many reviewers assessed each study and whether they worked independently, and if applicable, details of automation tools used in the process.	Discussion, Page 16
Effect measures	12	Specify for each outcome the effect measure(s) (e.g. risk ratio, mean difference) used in the synthesis or presentation of results.	Not applicable, qualitative data only
Synthesis methods	13a	Describe the processes used to decide which studies were eligible for each synthesis (e.g. tabulating the study intervention characteristics and comparing against the planned groups for each synthesis (item 5)).	Methods, Study selection, Page 19

(continued)

	13b	Describe any methods required to prepare the data for presentation or synthesis, such as handling of missing summary statistics, or data conversions.	Not applicable, qualitative data only
	13c	Describe any methods used to tabulate or visually display results of individual studies and syntheses.	Not applicable, qualitative data only
	13d	Describe any methods used to synthesize results and provide a rationale for the choice(s). If meta-analysis was performed, describe the model(s), method(s) to identify the presence and extent of statistical heterogeneity, and software package(s) used.	Not applicable, qualitative data only
	13e	Describe any methods used to explore possible causes of heterogeneity among study results (e.g. subgroup analysis, meta-regression).	Not applicable, qualitative data only
	13f	Describe any sensitivity analyses conducted to assess robustness of the synthesized results.	Not applicable, qualitative data only
Reporting bias assessment	14	Describe any methods used to assess risk of bias due to missing results in a synthesis (arising from reporting biases).	Discussion, Page 16
Certainty assessment	15	Describe any methods used to assess certainty (or confidence) in the body of evidence for an outcome.	Not applicable, qualitative data only
**RESULTS**			
Study selection	16a	Describe the results of the search and selection process, from the number of records identified in the search to the number of studies included in the review, ideally using a flow diagram.	Methods, Study selection and characteristics, Page 19-20
	16b	Cite studies that might appear to meet the inclusion criteria, but which were excluded, and explain why they were excluded.	Methods, Study selection and characteristics, Page 19-20
Study characteristics	17	Cite each included study and present its characteristics.	Methods, Study selection and characteristics, Page 19-20

(continued)

Risk of bias in studies	18	Present assessments of risk of bias for each included study.	Discussion, Page 16
Results of individual studies	19	For all outcomes, present, for each study: (a) summary statistics for each group (where appropriate) and (b) an effect estimate and its precision (e.g. confidence/credible interval), ideally using structured tables or plots.	Not applicable, qualitative data only
Results of syntheses	20a	For each synthesis, briefly summarise the characteristics and risk of bias among contributing studies.	Not applicable, qualitative data only
	20b	Present results of all statistical syntheses conducted. If meta-analysis was done, present for each the summary estimate and its precision (e.g. confidence/credible interval) and measures of statistical heterogeneity. If comparing groups, describe the direction of the effect.	Not applicable, qualitative data only
	20c	Present results of all investigations of possible causes of heterogeneity among study results.	Not applicable, qualitative data only
	20d	Present results of all sensitivity analyses conducted to assess the robustness of the synthesized results.	Not applicable, qualitative data only
Reporting biases	21	Present assessments of risk of bias due to missing results (arising from reporting biases) for each synthesis assessed.	Not applicable, qualitative data only
Certainty of evidence	22	Present assessments of certainty (or confidence) in the body of evidence for each outcome assessed.	Not applicable, qualitative data only
**DISCUSSION**			
Discussion	23a	Provide a general interpretation of the results in the context of other evidence.	Discussion, Page 16-18
	23b	Discuss any limitations of the evidence included in the review.	Discussion, Page 16-18
	23c	Discuss any limitations of the review processes used.	Discussion, Page 16-18

(continued)

	23d	Discuss implications of the results for practice, policy, and future research.	Discussion, Page 16-18
**OTHER INFORMATION**			
Registration and protocol	24a	Provide registration information for the review, including register name and registration number, or state that the review was not registered.	Methods, Literature Search and Study Selection, Page 19
	24b	Indicate where the review protocol can be accessed, or state that a protocol was not prepared.	Methods, Literature Search and Study Selection, Page 19
	24c	Describe and explain any amendments to information provided at registration or in the protocol.	Not applicable
Support	25	Describe sources of financial or non-financial support for the review, and the role of the funders or sponsors in the review.	Not applicable
Competing interests	26	Declare any competing interests of review authors.	Competing interests, Page 21
Availability of data, code and other materials	27	Report which of the following are publicly available and where they can be found: template data collection forms; data extracted from included studies; data used for all analyses; analytic code; any other materials used in the review.	Data availability, Page 21

PRIMSA Abstract Checklist

**TITLE**			
Title	1	Identify the report as a systematic review.	Yes
**BACKGROUND**			
Objectives	2	Provide an explicit statement of the main objective(s) or question(s) the review addresses.	Yes
**METHODS**			
Eligibility criteria	3	Specify the inclusion and exclusion criteria for the review.	Yes
Information sources	4	Specify the information sources (e.g. databases, registers) used to identify studies and the date when each was last searched.	No
Risk of bias	5	Specify the methods used to assess risk of bias in the included studies.	Yes
Synthesis of results	6	Specify the methods used to present and synthesize results.	Yes
**RESULTS**			
Included studies	7	Give the total number of included studies and participants and summarise relevant characteristics of studies.	No
Synthesis of results	8	Present results for main outcomes, preferably indicating the number of included studies and participants for each. If meta-analysis was done, report the summary estimate and confidence/credible interval. If comparing groups, indicate the direction of the effect (i.e. which group is favoured).	Yes
**DISCUSSION**			
Limitations of evidence	9	Provide a brief summary of the limitations of the evidence included in the review (e.g. study risk of bias, inconsistency and imprecision).	Yes
Interpretation	10	Provide a general interpretation of the results and important implications.	Yes
**OTHER**			
Funding	11	Specify the primary source of funding for the review.	Yes
Registration	12	Provide the register name and registration number.	No

From: Page MJ, McKenzie JE, Bossuyt PM, Boutron I, Hoffmann TC, Mulrow CD, et al. The PRISMA 2020 statement: an updated guideline for reporting systematic reviews. MetaArXiv. 2020, September 14. DOI: 10.31222/osf.io/v7gm2. For more information, visit: www.prisma-statement.org
